# Non-Digestible Oligosaccharides: A Novel Treatment for Respiratory Infections?

**DOI:** 10.3390/nu14235033

**Published:** 2022-11-26

**Authors:** Yang Cai, Gert Folkerts, Saskia Braber

**Affiliations:** 1 Department of Pharmacology, School of Medicine, Southeast University, Nanjing 210009, China; 2 Division of Pharmacology, Utrecht Institute for Pharmaceutical Sciences, Faculty of Science, Utrecht University, 3584 CG Utrecht, The Netherlands

**Keywords:** oligosaccharides, lung infections, respiratory inflammation, HMOs, GOS, FOS, gut microbiota, SCFAs

## Abstract

Emerging antimicrobial resistance in respiratory infections requires novel intervention strategies. Non-digestible oligosaccharides (NDOs) are a diverse group of carbohydrates with broad protective effects. In addition to promoting the colonization of beneficial gut microbiota and maintaining the intestinal homeostasis, NDOs act as decoy receptors, effectively blocking the attachment of pathogens on host cells. NDOs also function as a bacteriostatic agent, inhibiting the growth of specific pathogenic bacteria. Based on this fact, NDOs potentiate the actions of antimicrobial drugs. Therefore, there is an increasing interest in characterizing the anti-infective properties of NDOs. This focused review provides insights into the mechanisms by which representative NDOs may suppress respiratory infections by targeting pathogens and host cells. We summarized the most interesting mechanisms of NDOs, including maintenance of gut microbiota homeostasis, interference with TLR-mediated signaling, anti-oxidative effects and bacterial toxin neutralization, bacteriostatic and bactericidal effects, and anti-adhesion or anti-invasive properties. A detailed understanding of anti-infective mechanisms of NDOs against respiratory pathogens may contribute to the development of add-on therapy or alternatives to antimicrobials.

## 1. Respiratory Infections

Respiratory infections are the largest cause of childhood deaths [1,2] and an are important cause of morbidity and mortality among adults worldwide [3,4]. Respiratory infections take responsibility for approximately 4 million deaths per year globally [3]. Among them, lung infections are a common and potentially life-threatening illness, being a major medical burden accounting for more than 15% of the deaths of children younger than 5 years of age [2]. Risk factors for the incidence and severity of lung infections in infants and children mostly include the lack of immunization, malnutrition, chronic underlying diseases, HIV infection and smoke exposure/indoor air pollution [5]. The Global Burden of Disease Study indicated that the most important risk factors were malnutrition, air pollution or sub-optimal breastfeeding [6]. In 2015, although the improvement of living conditions, nutrition and vaccines, 700,000 children younger than 5 years of age still died from lung infections worldwide [6]. In particular, the COVID-19 pandemic has increased the emergency of taking action to protect against respiratory infections.

Transmission of lung infections is thought to occur by airborne droplets/pathogens or through direct contact with colonized/infected individuals. The epithelial mucosal surface of the lungs is constantly exposed to invasive pathogens that have the potential to threaten the defense of susceptible hosts [7]. After the epithelial mucosa is invaded by pathogens, the inflammatory response occurs subsequently to recruit additional defenses. However, when these pathogens have the capacity to overwhelm the host defense, invasion of pathogens results in infections of the respiratory tract [7,8,9].

## 2. Lung Epithelial Cells and Pro-Inflammatory Responses in Respiratory Infections

The well-developed respiratory defense is a dynamic interactive system against inhaled bacteria, including mucociliary clearance and pro-inflammatory responses of the respiratory epithelium, resident alveolar macrophages, and recruited neutrophils and lymphocytes [7,9]. Nevertheless, cold air and viral coinfection impair the mucociliary clearance, leading to a marked reduction in ciliary beat frequency and mucus transport velocity [9]. In addition, the secretion of bacterial toxins and the effect of bacterial attachment impede the ciliary function. For example, *Mycoplasma pneumoniae* expresses adhesins to achieve close interaction with host cells to resist mucociliary clearance [8,9]. Alternatively, nonspecific adherence to host cell surfaces via capsular polysaccharide or other bacterial proteins, like LPS and adhesins might occur as well, e.g., *Mannheimia haemolytica* (a ruminant respiratory pathogen) [10].

The respiratory epithelium is an important line of defense against inhaled pathogens through the physical barrier and its immunological functions [11]. Epithelial cells are connected to each other by cell–cell junctions, including tight junctions and adherens junctions, forming an impermeable mechanical barrier to prevent invasion of pathogens [7,11]. In addition to physical protection, lung epithelial cells possess important immune functions in the defense against pathogens. For instance, lung epithelial cells rapidly recognize pathogens through pattern recognition receptors (PRRs), such as Toll-like receptors (TLRs) and NOD-like receptors (NLRs) [7]. Upon PRR activation, lung epithelial cells not only generate cytokines/chemokines that recruit and activate leukocytes, but also produce endogenous danger signal molecules (e.g., ROS) [7,12].

Toll-like receptors (TLRs), such as TLR-2 and 4, recognize distinct pathogen-associated molecular patterns (PAMPs) derived from pathogens and detect damage-associated molecular patterns (DAMPs) released by stressed or damaged host cells [13]. These TLRs are expressed in airway epithelial cells and immune cells of both human and animals [9,13]. The recognition of *M. pneumoniae* by human airway epithelial cells and the activation of macrophages are dependent on TLRs, particularly TLR-2, which thereafter results in a subsequent inflammatory response such as IL-1 and IL-8 pro-inflammatory mediator release [8]. TLR-4, one of the well-characterized TLRs, senses LPS of bacteria and regulates pulmonary immunity to many Gram-negative pathogens [9,13]. LPS is recognized by TLR-4 on the surfaces of epithelial cells and alveolar macrophages, leading to the production of pro-inflammatory cytokines/chemokines, reactive nitrogen and oxygen intermediates, and other mediators. Subsequently, these pro-inflammatory cytokines/chemokines and mediators initiate an influx of neutrophils [10,14]. The cytokine/chemokine production and leukocyte activation may either minimize respiratory infections and eliminate the bacterial pathogens or, more often, exacerbate the disease through immunological hypersensitivity and worsening damage to the respiratory epithelium. More vigorous cytokine stimulation and cell-mediated responses lead to more severe lung injury [8].

Notably, a well-developed airway epithelial barrier is critical in the prevention of bacterial adhesions and invasions. The airway epithelium forms a complex physicochemical barrier complemented by the mucociliary escalator to provide the first line of defense against inhaled pathogens [11]. However, respiratory pathogens exhibit strategies to impair airway epithelium leading to pathogen invasion and colonization. Pathogenic bacteria may disrupt the airway epithelial integrity through their cytotoxicity or with the help of virulence factors, causing increased paracellular permeability and damaged epithelial repair mechanisms [8,9,11,15]. In addition, excessive inflammatory responses induced by pathogens also lead to a disrupted airway epithelial barrier [15].

Although the host has carefully designed the respiratory defense system, including a protective physical barrier, epithelial cell-mediated pro-inflammatory response, resident macrophages and recruited neutrophils, these defenses are susceptible to failure as a result of the presence of cofactors (e.g., stress, air pollution) and strategies adopted by pathogens (e.g., released virulence factors) [8,9,11]. Due to the failure of respiratory defenses, the strategies of pathogens, and the alarms of rising antimicrobial resistance, the development of add-on therapy or alternatives to antimicrobials for respiratory infections is particularly important.

## 3. Non-Digestible Oligosaccharides (NDOs)

NDOs are low molecular weight carbohydrates, usually containing 3 to 10 sugar moieties. Food products that naturally contain NDOs, such as cereals, fruits, vegetables, nuts, beans, and seafood, but also breast milk and the application of prebiotics in functional food are the main sources of NDO intake [16]. Many NDOs are not digested by humans due to the lack of enzymes required to hydrolyze the β-links formed among the monosaccharide units. The most famous physicochemical and physiological properties of NDOs are related to their ability to behave like dietary fibers and prebiotics, including the improvement of gut microbial composition and gastrointestinal homeostasis. Moreover, due to the decrease in intestinal pH caused by their fermentation, NDOs exhibit the ability to reduce the growth of pathogenic bacteria, increase the populations of *bifidobacteria* and *lactobacilli*, and increase the utilization of minerals in the gut [16]. In addition, NDOs are associated with a lower risk of (gastrointestinal, respiratory, and urogenital) infections and exert anti-inflammatory and immunomodulatory properties [16,17]. In this review, we mainly focused on several representative NDOs, including human milk oligosaccharides (HMOs), and galacto-oligosaccharides (GOS) and fructo-oligosaccharides (FOS) that mimic structures observed in mother milk.

### 3.1. HMOs

Human milk is the golden standard for infant nutrition, as most health experts, including the American Academy of Pediatrics, recommend exclusive breastfeeding for the first six months of life [18]. The average macromolecular profile of one liter of breast milk contains 9–12 g of proteins, 32–36 g of fats, 67–78 g of lactose, and 5–20 g of HMOs [19]. HMOs are the first group of NDOs consumed by humans after birth. Breastfed infants have a lower incidence of respiratory diseases, including respiratory infections, during early life [20,21,22,23]. In addition to the well-known prebiotic properties of HMOs and corresponding immunomodulatory effects [16], approximately 1–5% of HMOs are absorbed by the intestine into the systemic circulation [24], directly interacting with pathogens, immune cells, and epithelial cells outside the intestine to exert anti-inflammatory and anti-infective effects [16]. Moreover, breastfeeding infants ingest mother milk several times per day, bathing the nasopharynx and mouth for several minutes at each feeding with a solution high in HMOs, which might inhibit local adherence of airway pathogenic bacteria [20]. Bovine milk-derived infant formula is commonly used as an alternative to human breast milk. However, the concentration of oligosaccharides in bovine milk (0.7–1.2 g/L) is much lower compared to human milk (12–24 g/L) [25,26]. HMOs are composed of the five monosaccharide building blocks D-glucose, D-galactose, N-acetylglucosamine, L-fucose, and sialic acid. Currently, around 200 oligosaccharide structures have been identified in human milk compared to ± 50 identified oligosaccharide structures in bovine milk [27]. More than 70% of the oligosaccharides in bovine milk are composed of sialylated oligosaccharides, in contrast, human milk contains predominantly neutral oligosaccharides, and sialylated oligosaccharides account for approximately 10–30% of total HMOs [24,26,27,28,29]. Despite recent modern analytical techniques, the identification and biosynthesis of HMOs remain a challenge for researchers. The composition and content of HMOs can vary considerably between mothers. It depends both on their blood group and on the duration/length of the lactation period. Many manufacturers are trying to emulate HMOs; however, due to the lack of industrial production methods, the essential ingredients are mostly absent from infant formulas [30].

### 3.2. GOS and FOS

Various strategies have been used to mimic the beneficial effects of HMOs, including GOS and FOS, which have been supplemented in dietary products and infant formula [31,32]. Commercial production of GOS has been achieved from lactose by the transgalactosylation reactions, using β-galactosidases (EC 3.2.1.23) as biocatalysts [17,33]. FOS can be produced from the controlled enzymatic hydrolysis of the polysaccharide inulin, which can be extracted from roots of chicory, artichoke, yacon, dahlia or agave [33,34]. The process involves transfructosylation reactions in which fructosyltransferases (β-fructofuranosidase, EC 3.2.1.26 or β-D-fructosyltransferase, EC 2.4.1.9) act as biocatalysts [33]. GOS/FOS in a 9:1 ratio is commonly used in infant formula to mimic the molecular size distribution and beneficial functions of HMOs in breast milk [35].

**Figure 1:**
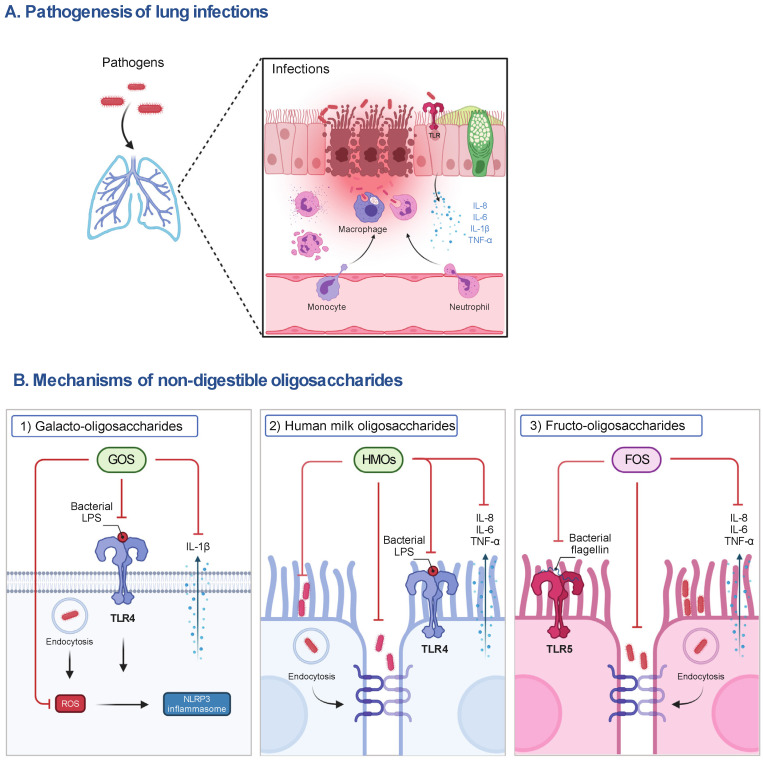
The pathogenesis of lung infections and the postulated mechanisms of representative NDOs on host cells. (**A**) During lung infections, pathogens can induce the release of pro-inflammatory mediators (e.g., IL-8, IL-6, IL-1β and TNF-α) by activating TLR signaling to recruit immune cells (e.g., neutrophils, macrophages), contributing to the phagocytosis of pathogens and elimination of inflammation in the early stage of infections. However, the impairment of the airway epithelial barrier, the accumulation of pro-inflammatory mediators, the depletion of macrophages, and the infiltration of neutrophils caused by excessive pathogens and their released virulence factors (e.g., LPS), lead to lung injury and organ dysfunction and even death of susceptible hosts. (**B**) (1) The anti-inflammatory mechanisms of NDOs (e.g., GOS) may include the inhibition of NLRP3 inflammasome activation via the interference with TLR-4 signaling and the decrease in ROS production, subsequently reducing IL-1β release, and (2) the decrease in adhesion to and invasion of airway epithelial cells by pathogens or the direct killing of pathogens induced by NDOs (e.g., HMOs). (3) Anti-inflammatory effects of NDOs (e.g., FOS) may be related to the interference with TLR-5 pro-inflammatory signaling and protection of airway epithelial barrier function. FOS, fructo-oligosaccharides; GOS, galacto-oligosaccharides; IL, interleukin; LPS, lipopolysaccharides; NLRP3, NLR family pyrin domain containing 3; ROS, reactive oxygen species; TLR, Toll-like receptor; TNF-α, tumor necrosis factor-α.

Both GOS and FOS exert many beneficial properties. For example, both can stimulate the growth of *bifidobacteria* and *lactobacilli* and support the development of the immune system. Moreover, both can inhibit the inflammatory responses and prevent epithelial barrier dysfunction in the intestine; in particular, GOS have the property of inhibiting the adhesion of pathogens to intestinal epithelial cells [16,32]. There is no doubt that GOS/FOS mixtures have similar properties, and even recently, a reduction in airway inflammation after oral administration of this mixture was demonstrated [36,37]. There are several studies investigating the supplementation of GOS and/or FOS in human respiratory infections mainly focusing on clinical parameters, observing a reduced frequency of respiratory infections and antibiotic prescriptions in infants, as well as a decreased duration and symptoms of cold or flu in university students [38,39,40]. These impressive observations encourage GOS and/or FOS to become attractive candidates in the prevention and clinical treatment of respiratory infections. Although not reported in respiratory infections, the effects of NDOs in some studies of inflammatory immune diseases are inconsistent; for example, a combination of probiotics and GOS showed no preventive effect on allergic diseases in infants [41]. In a study with mice, a mixture of FOS and inulin did not affect the immune response of delayed hypersensitivity in an influenza vaccination model [42]. In this focused review, we present a balanced overview of the role and mechanisms of NDOs in respiratory diseases (infections) (Figure 1).

## 4. Mechanisms of NDOs in Suppressing Respiratory Infections

### 4.1. Maintenance of Gut Microbiota Homeostasis

The main benefit of NDOs is to act as prebiotics, promoting the establishment of beneficial microbiota in the gut [16]. NDOs select and promote the growth of a few dominant valuable species, such as *Bifidobacterium* and *Lactobacillus spp.*, which competitively suppress the growth of pathogenic bacteria [16]. *Bifidobacteria* in infants have shown high incorporation with GOS, while FOS can selectively stimulate the growth of *Lactobacilli* [43]. In a double-blind controlled trial, administration of a selected *Lactobacillus fermentum* CECT5716 from human breast milk to infants aged 6–12 months reduced the incidence of gastrointestinal, respiratory and total infections by 46%, 27% and 30%, respectively [44]. HMOs in breast milk contribute to shape the gut microbiota in infants. Therefore, GOS and long-chain FOS are supplemented in infant formula at a ratio of 9:1 to mimic the function and size distribution of neutral HMOs in breast milk [38]. In a double-blind placebo-controlled trial, oral GOS and long-chain FOS at a ratio of 9:1 reduced the number of infectious episodes and the incidence of recurring, particularly respiratory, infections during the first 6 months of life [38]. In general, *Bacteroides*, *Lactobacillus*, and *Bifidobacterium* spp. can ferment NDOs in the gut, leading to the production of short-chain fatty acids (SCFAs; acetate, propionate, and butyrate). SCFAs protect the commensal bacteria against invading pathogens and inflammation by regulating gut pH and enhancing the immune system [16,19]. These SCFA molecules are small enough to diffuse through intestinal cells and enter blood circulation [45,46]. Sodium propionate, one of the SCFAs, was first described for potential bactericidal activity in 1950 [47]. Butyrate has been shown to increase the production of antimicrobial peptides in the lung epithelial cell line VA10 [48]. The importance of SCFA-acetate in protecting influenza-infected mice from secondary pneumococcal superinfection has been demonstrated [49]. Acetate treatment showed protective in pulmonary infections caused by *Klebsiella pneumoniae* in mice [50]. In addition, SCFAs affected the growth of *Pseudomonas aeruginosa* in vitro in a pH- and concentration-dependent manner [51]. Recently, Machado et al. have summarized the potential anti-infective effects of SCFAs in lung infections, including direct effects by inhibiting the growth of microbial pathogens and indirect effects by activating G protein-coupled receptors, inhibiting histone deacetylase, or interfering with metabolic pathways to alleviate lung infections [52]. Therefore, NDOs (and SCFAs) are able to affect infections not only in the gastrointestinal track but also in other distant site organs and systems.

### 4.2. Interference with TLR-Mediated Signaling

In addition to the indirect effects of NDOs on lung immunity via modulating gut microbiota homeostasis, increasing evidence shows that NDOs have the capacity to be absorbed into the systemic circulation (possibly in small amounts) after oral administration [53,54], indicating that NDOs might reach the lungs (bronchus) through the blood circulation to exert direct effects on pathogens or host cells (e.g., airway epithelial cells).

NDOs may act as a TLR ligand, affecting downstream NF-κB and cytokine/chemokine production [17]. We have reported that GOS can inhibit *M. haemolytica*-induced cytokine/chemokine production, TLR-4 expression and the associated MAPK/NF-κB pathway, as well as reduce the LPS (TLR-4 ligand)-induced cytokine/chemokine release in calf primary bronchial epithelial cells (PBECs) [55]. In addition, we found that FOS can inhibit the TLR-5-mediated pro-inflammatory pathway in calf PBECs and human A549 cells [56]. In studies with mouse splenocytes and rat small intestinal epithelial cells, pre-incubation with GOS for 30min or 1h inhibited LPS-induced pro-inflammatory responses [48,49]. This might be related to the interference of GOS with TLR-4-mediated signaling, downregulating TLR-4/NF-κB and subsequent MAPK pathways. HMOs, which exhibit similar anti-inflammatory effects as GOS, inhibit TLR-4 expression and signaling in mouse and piglet necrotizing colitis models and human (infant) intestinal explants, probably due to the capacity of HMOs to directly dock into the LPS-binding pocket of TLR-4 [50]. Comparable to the bacterial endotoxin LPS, GOS might competitively bind to TLR-4 of the lung epithelium to attenuate the initiation of the inflammatory response, thereby alleviating the airway inflammation caused by lung infections. Besides affecting epithelial cells, increasing studies have reported the immunomodulatory and anti-inflammatory effects of NDOs via the regulation of TLR signaling. For example, FOS may act as TLR-4 ligands to upregulate TNF-α and IL-10 secretion in primary rat monocytes and human peripheral blood monocytes but inhibited LPS -induced IFN-γ and IL-17 release in mouse splenocytes [57]. Regulation of macrophage immunomodulation via TLR4 by HMOs is dose and structure dependent. HMO 3-fucosyllactose can only activate TLR-2 and Lacto-N-Triaose activated TLR-2, 3, 4, 5, 7, 8 and 9 in a dose-dependent manner in HEK cells. Moreover, 2′-fucosyllactose, 6′-sialyllactose and Lacto-N-neotetraose inhibited TLR-5 and 7, while 3-fucosyllactose inhibited TLR-5, 7 and 8 in HEK cells [58]. In addition, wheatgrass-derived oligosaccharides activated monocytes via TLR-2 signaling [59], and feruloylated oligosaccharides induced DC maturation through TLR-2 and 4 [60]. However, caution should be taken, since modulation of inflammation is a delicate matter, and too much suppression may not benefit the host.

### 4.3. Anti-Oxidative Effect and Neutralization with Bacterial Toxins

NDOs, including inulin (long-chain FOS), raffinose and arabinoxylan-oligosaccharides, may act as reactive oxygen species (ROS) scavengers [61]. We demonstrated the anti-oxidative effect of GOS in vitro. GOS can inhibit the release of mitochondrial ROS and the activation of NLRP3 inflammasome in *M. haemolytica*-infected calf PBECs. Malondialdehyde level (a biomarker for oxidative stress) in blood of calves was inhibited by oral GOS [55]. NDO absorption into the systemic circulation may directly reduce the ROS production from host cells. On the other hand, the production of glutathione S-transferases induced by SCFAs, the fermentation products of NDOs in the gut, indirectly counteract ROS in vivo [61].

Respiratory pathogens can release virulence factors to induce oxidative stress and oxidative bursts in the lungs [14,62]. NDOs, such as GOS and HMOs, inhibit pathogen-induced cytotoxicity by neutralizing or interfering with released toxins [16]. Our group has reviewed the interactions between NDOs and bacterial toxins. NDOs and SCFAs can affect enterotoxins through several mechanisms, including metabolic integration, microbiota regulation, inhibition of fluid secretion, and maintenance of intestinal epithelial integrity [63]. Therefore, it is also possible that NDOs may indirectly inhibit the production of ROS by neutralizing or interfering with the toxins released by respiratory pathogens.

### 4.4. Bacteriostatic and Bactericidal Effects

Interestingly, some types of NDOs exhibit anti-bacterial effects [16,64]. Homogeneous and heterogeneous HMOs inhibited bacterial growth and biofilm assembly [65,66,67,68]. HMOs exert antibiofilm and antimicrobial properties against *Streptococcus agalactiae*, antimicrobial properties against the Gram-negative aerobe *Acinetobacter baumannii*, and antibiofilm properties against methicillin-resistant *Staphylococcus aureus*. Moreover, HMOs potentiate the action of aminoglycosides, antifolates, macrolides, lincosamides and tetracyclines against *S. agalactiae*, *S. aureus*, and *A. baumannii* [66,69]. In addition to serving as prebiotics, GOS possess bacteriostatic and bactericidal properties. GOS can not only inhibit *M. haemolytica* growth but also kill *M. haemolytica* in a concentration-dependent manner in vitro. The bactericidal effect of GOS might be related to the increase in bacterial membrane permeability as observed in GOS-treated *M. haemolytica* in vitro. GOS can affect the function of bacterial cell membranes through depolarization, and this may lead to the increased efficacy of antimicrobial drugs [70]. Similarly, HMOs increase the permeability of *S. agalactiae* cell membranes in a concentration-dependent manner to exert anti-bacterial activity [66,71].

### 4.5. Anti-Adhesion or Anti-Invasive Properties

NDOs (e.g., HMOs, GOS, FOS) can share structural homology with epithelial cell surface glycans, and serve as soluble decoy receptors to prevent early cellular attachment [19]. HMOs (lacto-N-neotetraose and α2-6-sialylated lacto-N-neotetraose) inhibit the adhesion of *pneumococci* to human lung epithelial cell line (A549) in vitro [72]. In vitro studies showed that the adherence of enteropathogenic *Escherichia coli* and *Cronobacter sakazakii* to intestinal epithelial cells can be inhibited by GOS [73,74]. GOS inhibited the adhesion to and invasion of PBECs by *M. haemolytica*, and therefore reduced inflammatory responses and airway epithelial barrier disruption [70]. NDOs additionally can indirectly prevent pathogen adhesion though binding to the epithelial surface, causing changes in receptor structure. These interactions include the carbohydrate–lectin interactions that play an important role in infections, as the cell surface of pathogens is decorated with oligosaccharides that recognize lectins on host cells [19].

## 5. Future Directions

NDOs with or without the combination of antibiotics might be a promising intervention to combat lung infections. Based on the described anti-infective mechanisms of NDOs against respiratory pathogens, we propose local administration (e.g., intranasal application, nebulized inhalation) for NDOs to treat/prevent respiratory infections [70]. We have already taken a step towards the application of NDOs against respiratory infections and GOS were innovatively applied via the intranasal route to ruminants (calf) to treat naturally occurring lung infections. Lower lung pathogen burden was observed in calves treated with an intranasal GOS spray [70,75]. Excitingly, our group started to investigate the effects of oral NDO intervention on airway diseases, such as asthma, chronic obstructive pulmonary disease (COPD), etc. years ago [36,76,77], and now showed that intranasal NDO administration might be the possible future strategy to inhibit respiratory infections [70]. Moreover, it has been described that infants who ingest breastmilk several times per day, bathing the nasopharynx for several minutes at each feeding with a solution high in HMOs, might inhibit local bacterial adherence [20]. However, the intranasal application of NDOs should be studied in more detail before being assessed in clinical trials or in practice. It will be of particular interest to investigate the changes in microbiota composition in the respiratory tract after intranasal administration of NDOs to unravel the exact mechanism by which NDOs exhibit anti-infective and anti-inflammatory properties.

The bacteriostatic properties of NDOs have brought new possibilities for the treatment of infections in a world of growing antimicrobial resistance. However, the bacteriostatic or even bactericidal mechanism of HMOs and GOS is still unclear. In the future, it may be interesting to study the effect of these antibacterial carbohydrates on bacterial glycocalyx, bacterial membrane formation and nutrient uptake. In addition, more carbohydrates should be included to study their (structure-related) anti-bacterial mechanisms. Furthermore, to increase our knowledge about the strain-specificity of GOS and other carbohydrates, more (human) pathogens should be involved in future research. Perhaps, it will be possible to design anti-pathogenic carbohydrates with corresponding structures based on different (drug-resistant) strains.

Besides the effect of NDOs on pathogenic bacteria as described above, the effect of NDOs on the commensal bacteria in the gut and airways during respiratory infections might also be interesting. It has been proven that the growth of commensal microbiota (e.g., *Bifidobacteria* and *Lactobacilli*) may inhibit the presence of pathogenic bacteria in the gastrointestinal and respiratory tract, which may be due to their competition for nutrition [16,75]. Due to the intervention of NDOs, the balance of nutritional competition may be biased towards the commensal microbiota.

The metabolic distribution of NDOs in the systemic circulation is not clear. Therefore, the presence of NDOs in blood and urine after different applications, as well as the metabolic kinetics of NDOs, should be measured. Moreover, it is already feasible and safe to supply oligosaccharides (such as αGal-oligosaccharides, glucose-oligosaccharides, and hyaluronic acid-derived oligosaccharides) via intravenous infusion in humans [78] and animals (e.g., mice, pigs, baboons) to provide energy sources or treat diseases [79,80,81]. This indicates the future possibility of intravenous application of GOS or FOS after a safety evaluation in humans and animals with respiratory diseases.

While the COVID-19 pandemic has been catastrophic, it will not be the last infectious disease that we have to deal with. The rapid functioning and globalization of modern society will lead to a disease spillover into humans around the world, which is far more facile than in the past. Humans and scientists must discover new interventions to face this challenge. Applications of NDOs in the treatment (or prevention) of respiratory infections are increasingly being investigated. Within this context, in our opinion, using NDO-based anti-infectives is a promising approach for the future, and the first clinical trial of using NDOs as antimicrobial alternatives or add-on therapy is likely on the horizon.

## Data Availability

Not applicable.
